# Behavioral and Socio-Emotional Disorders in Intellectual Giftedness: A Systematic Review

**DOI:** 10.1007/s10578-022-01420-w

**Published:** 2022-10-01

**Authors:** Ilaria Tasca, Michele Guidi, Patrizia Turriziani, Giovanni Mento, Vincenza Tarantino

**Affiliations:** 1 Department of Psychology, Educational Science and Human Movement, University of Palermo, Viale delle Scienze ed. 15, 90128 Palermo, Italy; 2 Servizio ULTREIA Cooperativa Progetto Insieme, via Cappello 42/44, 30027 Noventa Padovana, Italy; 3 General Psychology Department & Padova Neuroscience Center, University of Padua, via Venezia 8, 35131 Padua, Italy

**Keywords:** Giftedness, High intelligence, ADHD, Autism, Review

## Abstract

This work systematically reviewed past literature to investigate the association between intellectual giftedness and socio-emotional and/or behavioral disorders. Nineteen studies met the inclusion criteria, 17 of which have children and/or adolescents as participants, and 12 have a non-gifted control group. Socio-emotional problems, such as withdrawal, were found in 3 out of 8 studies; internalizing disorders, such as anxiety, were found in 5 out of 9; externalizing disorders, such as hyperactivity, were found in 3 out of 5. The most investigated comorbidity was attention-deficit/hyperactivity disorder. A univocal conclusion on the relationship between intellectual giftedness and socio-emotional/behavioral problems cannot be drawn, principally because of the heterogeneity of participants’ age, informants, and instruments. The review highlights the need for future studies to use multi-informant and comprehensive assessments, to reach more robust findings, and suggests that age and discrepancy between verbal and non-verbal intellectual abilities should be considered critical factors.

## Introduction

Numerous conceptions and countless definitions of giftedness have been put forth over the years. Renzulli was among the firsts to conceptualize giftedness[1]. According to the author, it reflects the interaction between three primary clusters (“rings”), namely, above-average ability, high levels of task commitment, and high levels of creativity. A gifted person shows “exceptional” performances in one or more specific areas, which can refer to different disciplinary fields (e.g., mathematics, science, technology) or transversal skills (e.g., communication, leadership, planning). Additionally, they show high levels of task commitment, that is, the energy that drives individuals to complete an undertaken activity, and creativity, that is, the expression of divergent and original thoughts.

Along with the seminal works of Renzulli, Heller et al. [2] proposed a model based on a psychometric classification. According to this model (the Munich Model of Giftedness), giftedness emerges from a network of relatively independent “talent” factors, namely, intelligence, creativity, social competence, musicality, artistic abilities, psycho-motor skills, and practical intelligence. The expression of these talent factors is moderated by non-cognitive/personality characteristics, such as achievement motivation, hope for success vs. fear of failure, thirst for knowledge, and ability to deal well with stress, as well as by socio-environmental conditions, such as educational style, parental demands on performance, social reactions to success and failure, number of siblings, family and school climate, quality of instruction, and critical life events. The interaction of factors and moderators converges in performance areas, such as mathematics, computer science, natural sciences, technology, languages, music, social activities, leadership, and athletics/sports.

Although giftedness emerges as a construct that goes beyond the mere correspondence with high intellectual abilities, it is often equated to high intelligence. Moreover, although the theories of multiple intelligences [3, 4] are endorsed the most, intellectual giftedness is still often equated with a high score on an intelligence quotient (IQ) test [5]. While addressing these limitations is beyond the scope of this review, we aimed the present work at investigating the relationship between high-IQ and the presence of socio-emotional and/or behavioral problems.

## Intellectual Giftedness

Intellectual giftedness is commonly identified by applying standardized measures of intelligence, such as the Wechsler Intelligence Scales (WPPSI/WISC/WAIS), which allow the estimation of individual IQ. In line with this approach, intelligence is a set of cognitive skills, related to planning, learning speed, reasoning, problem solving, understanding complex ideas, etc. [6]. The term “high intelligence” or “high intellectual potential” is usually attributed to those individuals who have reached or passed two standard deviations above the average IQ on the Wechsler Scales [7]. Based on IQ scores, in some countries, students may have access to special educational programs that help them address their special needs and maximally develop their potential, with enrichment and acceleration opportunities [see 8, 9 for a critical review].

Given the extraordinariness of high intelligence persons, the impact of intellectual giftedness on socio-emotional development has intrigued researchers [8]. However, empirical investigations have provided no consistent evidence of this impact. Some studies have found an association between giftedness and internalizing problems, which involve excessive control of emotions and behavior, anxiety, social withdrawal, low self-esteem, or excessive perfectionism [e.g., 11]. Other studies have instead found an association with externalizing behaviors, that is, actions acted under emotional dysregulation, hyperactivity, irritability, or aggressive behavior [e.g., 12]. On the other hand, high intellectual ability has been found also to be a protective factor against internalizing and externalizing difficulties, for both children and adolescents [5].

## Twice-Exceptionality

“Twice-exceptional” is the expression that refers to the condition in which giftedness is associated with a neuropsychiatric disorder. According to clinical records, attention-deficit/hyperactivity disorder (ADHD), autism spectrum disorder (ASD), and learning disabilities are the three diagnoses most often reported in comorbidity with giftedness [9, 10]. However, the differential diagnosis is complicated. Individuals with high intelligence may show typical ADHD-like features, such as high levels of activity, low impulse control, frustration and boredom, and poor attentional span [11]. According to Dąbrowski ([12] p. 303), giftedness is intended as “higher than average responsiveness to stimuli, manifested either by psychomotor, sensual, emotional (affective), imaginative, or intellectual excitability, or a combination thereof”. Therefore, the “psychomotor excitability” of a gifted person could be confused with the hyperactivity of ADHD, and the “imaginative excitability” could be interpreted as inattention. However, these problems diverge from those observed in individuals with ADHD with lower intelligence since they are not pervasive but context-dependent [13]. On the other hand, ADHD symptoms could be masked in gifted children due to their high IQ and successful classroom performance that offer helpful strategies for dealing with the problem [14].

Some gifted profiles might share socio-emotional problems with highfunctioning autism, mainly regarding social abilities, adaptation disorders, withdrawal in imaginary and/or intellectual abstraction, attention deficit, clumsiness, excessive attention to restricted interests, and hypersensitivity [7, 15, 16]. Nevertheless, although some typical social behaviors of autism spectrum disorder, particularly those falling in the range of Asperger’s syndrome, are similar to those exhibited by gifted children, the latter are keenly aware of the influence that their behavior might have on others and can soon understand the appropriateness of behaviors. Additionally, gifted children are often involved in community projects and can show leadership skills [17]. Conversely, children with Asperger’s have great difficulty understanding the perspective of others and fail to grasp the social behaviors that other children intuitively learn, being unaware of social conventions [18, 19]. On the other hand, gifted individuals could be socially isolated because of problems in meeting intellectual peers [7].

Regardless of the specific diagnostic profile, the question of whether high intelligence might represent a risk or a protective factor for developing socio-emotional and/or behavioral disorders remains. Francis and colleagues [5] addressed this issue and revealed that gifted people show better socio-emotional adjustments and fewer behavioral problems than their peers. However, the authors found that the association between intellectual giftedness and psychopathology greatly varies across studies. In line with the work of Francis and colleagues, the present study aimed at examining how often giftedness coexists with socio-emotional and behavioral disorders. Noteworthy, while Francis et al. [5] reviewed studies published before 2014, we narrowed the search to the last two decades, from 2000 to 2020. This allowed us to gather more recent empirical evidence to understand intellectual giftedness. Furthermore, we focused on the type of instruments used to assess the presence of such disorders, to unveil the potential confounding role of methodological factors in explaining inconsistent findings in the prevalence of socio-emotional and behavioral problems.

## Methods

This systematic review was performed in accordance with the Preferred Reporting Items for Systematic Reviews and Meta-Analyses (PRISMA) guidelines [20].

### Eligibility Criteria

We included studies examining socio-emotional and/or behavioral problems in intellectual giftedness using standardized instruments, such as questionnaires, check-lists, or interviews. Studies published in peer-reviewed journals and with publication dates from 2000 to 2020 were eligible. No restrictions on participants’ age were applied and only studies published in English were considered.

### Search Strategy

The literature search was conducted on two databases, PubMed and Web of Science. Keywords related to “intellectual giftedness” and “behavioral problems” were used in the database query. The following string was specifically entered: ((gifted OR “intellectual gifted*” OR “high intelligen*” OR “high ability”) AND IQ AND (disorder* OR problem*) AND (behav* OR social OR emotion* OR internalizing OR externalizing OR withdrawal OR hyperactivity OR attention OR ADHD OR autism OR ASD OR “learning disabilit*” OR DSA OR anxiety OR depress* OR (twice AND exceptional*))). Keywords were searched in the manuscripts’ title, abstract, and text. Additionally, the reference lists of the resulting articles and reviews were inspected to identify other relevant studies.

### Selection Process

**Fig. 1:**
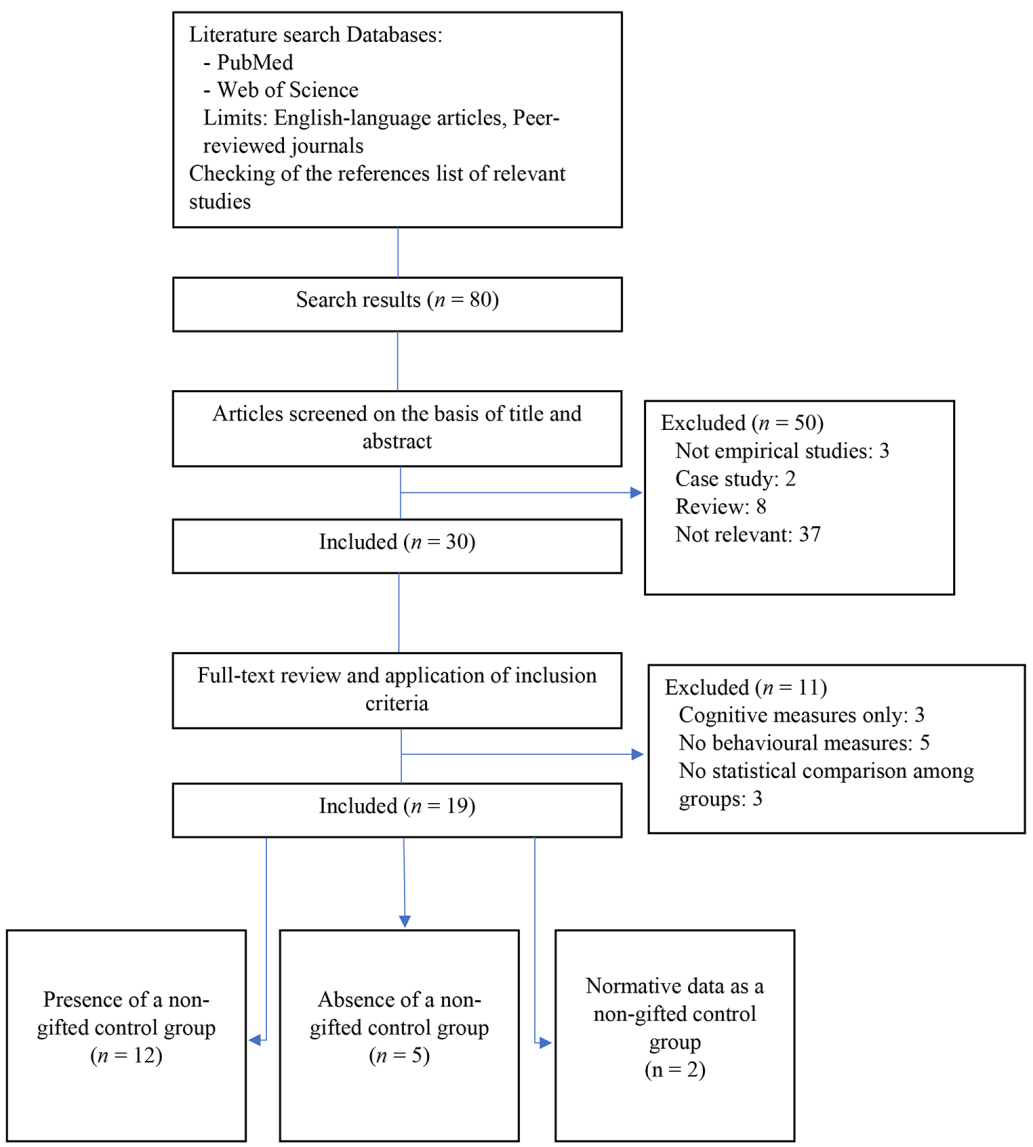
Flow-chart of the screening and selection processes

Figure 1 displays the flow chart of the study selection process. Of the 80 results of the literature search, 50 articles were selected based on the title and abstract. Non-empirical studies, case studies, reviews and studies that include material-specific giftedness were excluded in this first selection. Afterward, manuscripts were reviewed and included if providing full IQ, measures of socio-emotional or behavioral problems assessed by standardized tools, and statistical comparison among groups of participants. Moreover, studies that only provided cognitive measures, that did not state the measures used for the full IQ estimation and for the behavioral assessment, were excluded. Two authors (I.T. and V.T.) independently screened titles, abstracts, and full-text of the retrieved articles and applied the study’s inclusion and exclusion criteria. Any disagreement between the two authors was resolved through discussion.

From the review of manuscripts and the application of inclusion criteria, a total of 19 studies met the inclusion criteria. All the included studies have participants with a full IQ ≥ 120.

For each selected study, participants’ characteristics, control group, recruitment setting, behavioral measures, and outcomes are summarized in Table 1. Cognitive measures and outcomes are also provided in Table S1.

**Table 1:** Characteristics of the selected studies

AUTHORS	PARTICIPANTS	Non-gifted CONTROL GROUP	RECRUITMENT	COUNTRY	GIFTEDNESS CUT-OFF	BEHAVIORAL ASSESSMENT	BEHAVIORAL OUTCOMES				
Antshel et al. (2007)	2 groups: gifted (n = 92; 34% females; M.age = 11.6 y, SD = 3.6); gifted with ADHD (n = 49; 29% females; M.age = 10.6 y, SD = 3.4).	No	Massachusetts General Hospital Longitudinal Family Studies of ADHD; Pediatric Psychopharmacology Clinic; Harvard Community Health Plan.	America	IQ ≥ 120	K-SADS-PL (Parents); SAICA (Parents); CBCL (Parents).	CBCL: compared to the gifted group without ADHD, the gifted group with ADHD had higher scores in all subscales.	SAICA: compared to the gifted group without ADHD, the gifted group with ADHD reported more problems at school, in spare time, with peers, with the opposite sex, and with parents. Furthermore, the gifted group with ADHD carried out more activities in spare time and with father.	K-SADS: compared to the gifted group without ADHD, the gifted group with ADHD reported more symptoms of mood disorders (i.e., major depressive, bipolar), anxiety disorders (i.e., generalized anxiety phobias, social phobia, separation anxiety), and disruptive behavior disorders (oppositional defiant, conduct). The disorders with the highest prevalence were major depressive and oppositional defiant.		
Antshel et al. (2008) Follow-up of Antshel et al., 2007	2 groups: gifted (n = 79; 46% females; age range = 10–24 y, M.age = 16.4); gifted with ADHD (n = 49; 51% females; age range = 10–24 y, M.age = 16.9).	No	Massachusetts General Hospital Longitudinal Family Studies of ADHD; Pediatric Psychopharmacology Clinic; Harvard Community Health Plan.	America	IQ ≥ 120	K-SADS-PL (Parents); SAICA (Parents); CBCL (Parents).	CBCL: compared to the gifted group without ADHD, the gifted group with ADHD had higher scores in all subscales, except “thought problems”.	SAICA: compared to the gifted group without ADHD, the gifted group with ADHD has more problems at school, in spare time, with peers, with the opposite sex, and with parents. Furthermore, the gifted group with ADHD carried out more activities in spare time.	K-SADS: compared to the gifted group without ADHD, the gifted group with ADHD reported more symptoms of mood disorders (i.e., major depressive), anxiety disorders (i.e., phobias, social phobia, separation anxiety), and disruptive behavior disorders (oppositional defiant).		
Antshel et al. (2009)	2 groups: gifted (n = 53; 49% females; age range = 18–55 y, M.age = 27.9); gifted with ADHD (n = 64; 44% females; age range = 18–55 y, M.age = 33.4).	No	Psychiatric clinics; advertisements in the city area.	America	IQ ≥ 120	Q-LES-Q (Self-report); SAS-SR (Self-report); SCID (Parents).	Q-LES-Q: compared to gifted group, gifted group with ADHD had lower scores in mood, work, household activities, social relationships, family relationships, leisure time, daily life functioning, economic status, living/housing situation and overall life satisfaction.	SAS-SR: compared to the gifted group, the gifted group with ADHD reported being less likely to connect with friends, to talk with friends about their feelings/problems, and were more likely to have their feelings hurt by friends, feel lonely and feel bored. Conversely, they were just as likely as controls to go out socially with others, spend time on hobbies/interests, get in arguments with friends, and feel shy/uncomfortable with others.	SCID: compared to the gifted group, the gifted group with ADHD showed highest prevalence of major depressive disorders, obsessive-compulsive disorder, and generalized anxiety disorder.		
Chae, Kim, & Noh (2003)	2 groups: gifted (n = 106; 10 ADHD; 33 females; age range = 6–9 y, M.age = 7.7); non-gifted (n = 71; no of ADHD not specified; 31 females; age range = 6–9 y, M. age = 7.7).	Yes	Educational institute for gifted children	Korea	IQ ≥ 130	CBCL (Parents)	CBCL: social skills of gifted children, with and without ADHD, were rated significantly poorer than non-gifted children; gifted children with ADHD were poorer that gifted children without ADHD.				
Chung et al., (2011)	2 groups: gifted (n = 22; 6 females; age range: 13–15 y, M.age = 14); non-gifted (n = 26; 11 females; age range: 13–14 y, M.age = 14).	Yes	Private institute for special education for the gifted	Korea	IQ > 130	Public goods (PG) game	The two groups had the most significant cooperation difference in condition in which cooperation was rewarded, with the gifted group showing significantly higher cooperation.				
Doobay, Foley-Nicpon, Ali, & Assouline (2014)	2 groups: gifted (n = 41; 20 females; age range = 5–17 y, M.age = 9.4); gifted with ASD (n = 40; 6 females; age range = 5–17 y, M.age = 10.7).	No	University-based clinic	America	IQ ≥ 130	BASC-2 (Parents, Teachers, Self-report); Vineland-II (Parents).	BASC-2 parents: compared to gifted group without ASD, the gifted group with ASD had lower mean scores on the ASI, whereas they had higher scores on the BSI, and higher scores on the aggression, attention problems, atypicality, hyperactivity, somatization, and withdrawal scales.	BASC-2 teachers: compared to gifted group without ASD, the gifted group with ASD had higher mean scores on the BSI, and higher scores on the aggression, attention problems, atypicality, conduct problems, hyperactivity and the withdrawal scales.	BASC-2 self-report: the gifted group with ASD had higher mean scores on the ESI than gifted group without ASD.	Vineland-II: compared to gifted group without ASD, the gifted group with ASD had lower scores in the communication, daily living skills, and socialization domains.	
Eren, Cete, Avcil, & Baykara (2018)	2 groups: gifted (n = 49; 22 females; age range = 9–18 y); non-gifted (n = 56; 25 females; age range = 9–18 y).	Yes	Academic Science Art Center	Turkey	IQ ≥ 130	PedsQL (Children); SDQ (Parents, Children); STAI-C (Children); CDI (Children); CDRS-R (Clinician).	PedsQL: the gifted group obtained lower scores than the non-gifted group.	SDQ child: the gifted group had a significant higher score on inattention/ hyperactivity subtest. Males reported more frequent peer problems, whereas females reported higher social behaviors.	SDQ parent: no significant difference between groups.	STAI and CDI: no significant difference between groups.	CDRS-R: no significant difference between groups; males in the gifted group had higher depressive scores than females in gifted group.
Foley-Nicpon et al. (2012)	2 groups: gifted (n = 58; 20 females; age range = 6–16 y); gifted with ADHD (n = 54; 18 females; age range = 6–18 y).	No	Psychology clinic	America	IQ ≥ 120	BASC-2 (Children); PH-2 (Children).	BASC-2: The gifted group without ADHD reported higher positive self-esteem than the gifted group with ADHD. No differences between the two groups were found with regards to their perceptions of relationships with others, self-reliance, or social stress.			PH-2: The gifted group without ADHD reported higher self-concept about their behavior and greater overall happiness, compared to the gifted group with ADHD. No differences were found regarding their perceptions of their intelligence, physical attributes, self-reported symptoms of anxiety, or popularity.	
Gomez et al. (2019)	4 groups: gifted (n = 15; 5 females; M.age = 12.1 y, SD = 3.11); non-gifted (n = 124; 50 females; M.age = 10.8 y, SD = 3); gifted with ADHD (n = 18; 5 females; M.age = 10, SD = 3.7); non-gifted with ADHD (n = 350; 88 females; M.age = 10. 5, SD = 3).	Yes	Academic Child Psychiatry Unit	Australia	IQ ≥ 120	SWAN (Parents)	SWAN: Both gifted and non-gifted groups with ADHD had higher average scores (lower performance) in inattention, hyperactivity/impulsivity, and total ADHD scores compared to the two gifted and non-gifted groups without ADHD. The non-gifted group with ADHD reported higher scores than the gifted group with ADHD for all inattention symptoms, while for some hyperactivity/impulsivity symptoms (motor activity, verbal activity and reflect on question), the picture was reversed. The two groups without ADHD did not differ from each other in inattention, hyperactivity/impulsivity, and total ADHD scores.				
Guénolé et al. (2013)	2 groups: gifted (n = 143; 114 low-gifted; 29 high-gifted; 42 females; age range = 8–11 y, M.age = 9.3); non-gifted (n = 144; 42 females; age range = 8–11, M.age = 9.3).	Yes	Department of child and adolescent psychopathology; private practice pediatricians.	Australia	IQ ≥ 130	CBCL (Parents)	CBCL: the gifted group reported higher scores in all subscales, compared to the non-gifted group. The high-gifted subgroup reported higher percentages of externalizing disorders compared to the low-gifted subgroup (34.5% versus 14%), whereas the low-gifted subgroup reported higher percentage of mixed syndrome (24.5% versus 6.9%).				
Guignard, Jacquet, & Lubart (2012)	3 groups: gifted 6th graders (n = 61; M.age = 10.9 y, SD = 0.8); non-gifted 6th graders (n = 51; M.age = 11.6 y, SD = 0.6); non-gifted 5th graders (n = 20; M.age = 113 y, SD = 0.4).	Yes	Special classes	France	IQ ≥ 130 on at least one of the Index scores from the Weschler’s intelligence scales.	R-CMAS (Children); CAPS (Children).	R-CMAS: the 6th graders gifted group showed higher scores on Worry/Overexcitability and Social Concerns compared to 5th graders non-gifted group.			CAPS: the 6th graders gifted group had higher scores than the 6th graders non-gifted group on the Total Score and on self-oriented perfectionism.	
Karpinski et al. (2018)	2 groups: gifted (n = 3715; 1472 females; age range = 18–91 y, M.age = 53, SD = 15.2).	National average	American Mensa, Ltd., society	America	IQ ≥ 130 (as reported in a previously collected large dataset).	Online survey (participants were asked to indicate whether they have been either diagnosed or suspected they should be diagnosed with a variety of disorders).	The gifted group showed a higher number of diagnoses of all psychological disorders (mood, anxiety, ADHD, ASD) relative to the national average. As regards ASD, an increase of diagnoses was observed when considering the broad DSM-5 category as well as when isolating the Asperger’s Syndrome (DSM-IV).				
Kermarrec, Attinger, Guignard, & Tordjman (2020)	2 groups: gifted (n = 211; 36 females; age range = 6–11 y, M. age = 10.7); non-gifted (n = 397; 64 females; age range = 6–11 y, M. age = 10.5).	Yes	National Center for Assistance to children and adolescents with High Potential (CNAHP)	France	IQ ≥ 130	R-CMAS (Children); psychiatric and parental evaluation.	R-CMAS: scores on total anxiety, physiological anxiety and worry/oversensitivity were significantly higher in the VCI gifted group (VCI ≥ 130) compared with the VCI non-gifted group (VCI < 130); conversely, they were significantly lower in the PRI gifted group (PRI ≥ 130) compared with the PRI non-gifted group (PRI < 130).	Parents’ observation: no significant differences between groups.	Psychiatrist’s DSM-5 evaluation: anxiety disorders were diagnosed more often in the gifted group.		
Lacour & Zdanowicz (2019)	2 groups: gifted (n = 15; age range: 12–16 y); non-gifted (n = 20; age range: 12–16 y).	Yes	Specialized associations, social networks on the Internet.	France	IQ > 130 on at least one of the Index scores from the WISC-IV.	K-SADS-PL; SRAS-R (Adolescents); FACES III (Adolescents).	K-SADS-PL: the gifted group compared to the non-gifted group had fewer phobias, and thus less avoidance behaviors, was more shy, and had fewer friends, both in the real world and in the virtual world.	SRAS-R: the gifted group had fewer friends in real life, fewer virtual friend, and fewer virtual relations than the non-gifted group.		FACES III: shy gifted adolescents had a greater cohesion in their family than non-shy gifted adolescents.	
McCoach, Siegle, & Rubenstein (2020)	2 groups: gifted (n = 212; 25% female; age range: 9–17 y, M.age = 12).	Non-gifted normative sample for the ADHD-IV rating scales	National Association for Gifted Children	America	IQ ≥ 120	ADHD-IV rating scales (Parents, Teachers); SAAS-R (Adolescents).	ADHD-IV rating scales: higher scores in the gifted group for inattention versus hyperactivity, according to both parents and teachers; inattentive behaviors were reported more frequently in school settings than in home settings.			SAAS-R: gifted with elevated home inattention scores had lower scores on SAAS-R self-efficacy, goal valuation, and self-regulation subscales. The largest difference, compared with non-gifted group, emerged on the self-regulation subscale.	
Peyre et al. (2016)	3 groups: gifted (n = 23; age range = 5–6 y, M.age = 67.9 months); non-gifted (n = 1058; age range = 5–6 y, M.age = 67.9 months); IQ < 70 (n = 19; age range = 5–6 y, M.age = 67.9 months).	Yes	EDEN prospective mother–child cohort	France	IQ > 130	SDQ (Parents)	SDQ: no significant differences were found in behavioral, emotional and social problems between gifted and non-gifted groups; only one specific item (“many worries or often seems worried”) was significantly more frequent in gifted group.				
Richards, Encel, & Shute (2003)	2 groups: gifted (n = 33; 12 females; grades 7–10; M.age = 14.5); non-gifted (n = 25; 11 females; grades 7–10; M.age = 14.6).	Yes	College catering for students of all academic abilities	Australia	IQ ≥ 127 on at least one of the cognitive assessment measures.	BASC (Parents, Teachers, Children)	BASC parents: the mean score on the BSI of the gifted group was significantly lower than that of the non-gifted group, indicating better overall psychological adjustment in the gifted group. The gifted group scored lower on the Anxiety Scale and on Attention problems than the non-gifted group.	BASC Teachers: no significant differences were found between the two groups.	BASC self-report: the gifted group showed better adjustment than non-gifted group, that is, fewer depressive symptoms, a better attitude towards teachers, greater self-reliance and a greater sense of adequacy.		
Rommelse et al. (2017)	n = 2221; age range = 10–12 y	Yes	General population cohort study	Netherlands	IQ > 130	CBCL (Parents); TRF (Teachers); YSR (Self-report).	CBCL: higher IQ scores were associated with lower levels of attention and hyperactivity-impulsivity problems.	TRF: higher IQ score was related to lower levels of attention problems and hyperactivity-impulsivity problems.	YSR: higher IQ score was related to lower levels of attention problems and hyperactivity-impulsivity problems.		
Shaywitz et al. (2001)	4 groups (boys): low-gifted (n = 17; grades 4–7; M.age = 11.6 y); high-gifted (n = 18; grades 4–7; M.age = 11.1 y); learning disabilities (n = 26; grades 4–7; M.age = 11.5 y); non-gifted (n = 26; grades 4–7; M.age = 11.3 y)	Yes	Special program for gifted students	America	IQ > 130	YCI (Parents)	YCI: no significant differences between the gifted and the non-gifted group in withdraw/depressive symptoms, in attention problems, or in externalizing behaviors. The gifted group reported more negative affect than the non-gifted group. The high- vs. and low-gifted subgroups did not significantly differ. The learning disabilities groups were poorer in both behavioral and cognitive scale relative to the low-gifted group, and in the behavioral scale only relative to the high-gifted group.				

ADHD = Attention Deficit Hyperactivity Disorder; ADI-R = Autism Diagnostic Interview-Revised; ADOS = Autism Diagnostic Observation Schedule; ASD = Autism Spectrum Disorder; ASI = Adaptive Skills Index; BASC = Behavior Assessment System for Children; BSI = Behavioral Symptoms Index; CAPS = Children and Adolescent Perfectionism Scale; CBCL = Child Behavior Checklist; K-SADS = Kiddie-Schedule for Affective Disorders and Schizophrenia; CDI = Child Depression Inventory; CDRS-R = Children Depression Rating Scale Revised; ESI = Emotional Symptoms Index; FACES III = Family Adaptability and Cohesiveness Evaluation Scale III; FAD = Family Assessment Device; IQ = full IQ; M.age = mean age; PedsQL = Pediatric Quality of Life Inventory; PMAs = Primary Mental Abilities; PRI = Perceptual Reasoning Index; PSI = Processing Speed Index; Q-LES-Q = Quality of Life Enjoyment and Satisfaction Questionnaire; R-CMAS = Revised Children and Adolescent Manifest Anxiety Scale; SAAS-R = School Achievement Attitudes Survey-Revised; SAICA = Social Adjustment Inventory for Children and Adolescents; SAS-SR = Social Adjustment Scale Self-Report; SCID = Structured Clinical Interview for DSM-IV; SD = standard deviation (when present); SDQ = Strength and Difficulties Questionnaire; STAI-C = State-Trait Anxiety Inventory for Children; SRAS-R = School Refusal Assessment Scale-Revised; SWAN = e Strengths and Weaknesses of ADHD-Symptoms and Normal Behavior Scale; TRF = Teacher’s Report Form; VCI = Verbal Comprehension Index; Vineland-II = Vineland Adaptive Behavior Scales-Second Edition; WAIS-III = Wechsler Adult Intelligence Scale-3rd Edition; WISC-R = Wechsler Intelligence Scale for Children-Revised edition; WISC-III = Wechsler Intelligence Scale for Children-3rd Edition; WISC-IV = Wechsler Intelligence Scale for Children-4rd Edition; WJII = Woodcock-Johnson; WMI = Working Memory Index; WPPSI-III = Wechsler Preschool and Primary Scale of Intelligence 3rd Edition; WRAT-R = Wide Range Achievement Test-Revised edition; YCI = Yale Children’s Inventory; YSR = Youth Self-Report.

### Participants

The majority of studies involved children or adolescents aged between 4 and 18 years; only three studies involved adults, up to 24 [19], 55 [25], or 91 years [26]. In most of them (n = 12), “gifted” and “non-gifted” groups were compared. In two cases [26, 27] the gifted group was compared to the normative sample. In addition, some researchers performed comparisons between gifted participants with high (“low-gifted”) and very high (“high-gifted”) IQ [28, 29], or included an additional group with intellectual disability [30] or a learning disability [28] .

Participants were mainly enrolled in special programs for gifted children/adolescents [28, 31–35], whereas some of them were recruited in hospitals/clinics [19, 25, 29, 36–40], or from specialized associations for gifted children [27, 41]. Only a few of them enrolled participants from the general population [30, 42]. In one study [26], the gifted group was enrolled by the MENSA, an internationally recognized association grouping people with a verified IQ score within the top 2% of the general population.

### Cognitive and Behavioral Measures

The Wechsler’s intelligence scales [43–47] have been used as the main instruments to measure IQ. Other additional intelligence tests were sometimes performed (see Table S1 of Supplementary material for more information).

Various tools have been used for behavioral assessment, principally questionnaires, check-lists, or semi-structured interviews. Importantly, they could be addressed to parents, teachers, clinicians, and/or directly to children/adolescents. Most studies have used scales that provide a multi-dimensional behavioral assessment, revealing the frequency of problematic behaviors in multiple areas. All instruments used in the reviewed studies are listed and described in Supplementary materials. Among them, the most used were: the Child Behavior Checklist (CBCL [48]), whose subscales are grouped in those that investigate “internalizing” symptoms, those that investigate “externalizing” symptoms, those that investigate both (“mixed syndrome”), as well as items on social, thought and attention problems; the Behavior Assessment System for Children (BASC [49], BASC-2 [50]), which assesses externalizing, internalizing, attention, learning (for teachers), atypicality, and withdrawal problems; the Schedule for Affective Disorders and Schizophrenia for School Age Children, Present and Lifetime Version (K-SADS-PL [51]), which investigates the presence of multiple psychopathologies (e.g., depression and ADHD) according to DSM-IV criteria; the Strengths and Difficulties Questionnaire (SDQ [52]), wich measures pro-social behaviors, emotional symptoms, conduct problems, inattention/hyperactivity symptoms and peer relationship/problems; and the Revised Children and Adolescent Manifest Anxiety Scale (R-CMAS [53]) for the evaluation of the level and nature of anxiety through three subscales (physiological anxiety, worry/oversensitivity, and social concerns/concentration).

## Results

For the sake of simplicity, we grouped behavioral results into three broad areas: socio-emotional (e.g., withdrawn, attitude toward peers), internalizing (e.g., anxiety, negative affect), and externalizing (e.g., hyperactivity/impulsivity, conduct disorders) symptoms. Overall, three studies [29, 34, 41] out of 8 that investigated socio-emotional aspects found a higher presence of problems in this area in the gifted compared to the control group. Five studies [26, 28, 29, 34, 38] out of 9 that examined internalizing disorders, and three studies [28, 29, 33] out of 5 that examined externalizing disorders, found a higher prevalence of problems in the gifted group. Therefore, robust evidence that supports the presence or absence of an association between intellectual giftedness and behavioral disorders does not emerge. In most cases (n = 12), the control group included age-matched, within-average IQ participants. Two of them further compared low vs. high-gifted [28, 29]. One study added a group with learning disabilities [28], whereas another study added a group with intellectual disability [30]. One study analyzed the effect of IQ considered as a continuous variable [42]. Interestingly, one study uses two non-gifted control groups: a group that matched the grade level, and a group of a lower grade that matched age [34]. In some cases, twice-exceptionality was investigated, namely, gifted participants with an ADHD (n = 5) or an ASD (n = 1) diagnosis. With respect to age, eight studies examined preschool or school children, six studies examined both children and adolescents, three studies examined adolescents only, and two studies examined adults.

### Socio-Emotional Problems

Eight studies investigated socio-emotional problems in intellectual giftedness. Among the four studies involving children, two studies [29, 34] revealed the presence of socio-emotional problems in the gifted group. Two studies focused on adolescents, and one of them [41] found socio-emotional problems. One study [33], which was addressed to both children and adolescents, did not reveal the presence of problems related to the socio-emotional area. Finally, only one study [26] investigated adults and found no socio-emotional difficulties. More details are provided below.

Peyre et al. [30] investigated the presence of socio-emotional problems, reported on the parent-rated SDQ, by comparing gifted and non-gifted preschool children (5–6 years old) recruited in a general population mother-child cohort. They did not find between-group differences in items investigating peer relationships (e.g., isolation and ability to relate to peers compared to adults) and prosocial behaviors (e.g., consideration of others, willingness to comfort others). Using the SDQ, the authors investigated the presence of internalizing and externalizing problems as well. No significant differences with the control group emerged again. This study suggests that children with high IQ, recruited in the preschool age among the general population, did not show problematic behaviors. In contrast, the study of Guénolé et al. [29], which compared scores on parent-rated CBCL of clinically-referred gifted school children, aged 8 to 12, to scores of age- and sex-matched group of children recruited at school, revealed that gifted children displayed increased behavioral problems in the whole symptomatic range, including withdrawn and social domains.

Eren et al. [33] assessed social behaviors in gifted children and adolescents (9–18 years) attending a special school, and in a control group of participants of a regular school, using multiple instruments, namely, the K-SADS-PL, the SDQ, and the Pediatric Quality of Life Inventory (PedsQL; [54]), completed by parents and children (adolescent only in the case of SDQ). No significant differences between groups were found, in parents’ and children’s scores. Similarly, the group of gifted children and pre-adolescents of Guignard et al. [34], recruited at school with special classes, did not differ from the non-gifted control group, matched for education grade, in the “social concerns” score of the R-CMAS. Nevertheless, they exhibited significantly more social concerns than the non-gifted control group matched for age.

Lacour & Zdanowicz [41] found that gifted adolescents between 12 and 16 years reported having fewer friends in real life and fewer virtual friends compared to a non-gifted group, as assessed by the K-SADS-PL and the School Refusal Assessment Scale-Revised [55, 56]. The majority of them considered themselves shy, unsure of themselves, and experienced fear of social situations. Furthermore, they reported more irritability, negative affect, distractibility, and separation anxiety. On the other hand, they reported higher family cohesion in the Family Adaptability and Cohesiveness Evaluation Scale III [57] questionnaire. The authors attributed this latter evidence to a potential disincentive for them to open up and decrease their shyness.

On the other hand, Chung et al. [32] found a significantly higher cooperative tendency in gifted adolescents compared to non-gifted adolescents in a multi-player social interaction game, especially when cooperation was rewarded.

Karpinski et al. [26], using an online survey, asked gifted adult participants to indicate if they had been diagnosed with one or more disorders among a list (bipolar disorder, depressive disorder, generalized anxiety, social anxiety, obsessive-compulsive disorder, Asperger’s syndrome). Then, they compared the responses with those obtained from previous national surveys. A higher prevalence of disorders emerged in the gifted group. Social anxiety was the only domain in which differences were not found.

### Internalizing Problems

Internalizing problems were investigated the most (9 studies out of 19). Of these studies, six involved children, four of which [28, 29, 34, 38] revealed the presence of internalizing problems in the gifted group; two studies [33, 41] involved adolescents as well and found no internalizing problems. The study that involved adults [26] found an association between intellectual giftedness and internalizing problems.

In 5–6 years old children, Peyre et al. [30] found no association between intellectual giftedness and anxiety or depressive symptoms, rated by parents in the SDQ. Guignard et al. [34] assessed anxiety and perfectionism in gifted children in 6th grade, using the self-rated R-CMAS in conjunction with the Children and Adolescent Perfectionism Scale (CAPS; [58]). It turned out that gifted children did not differ from the non-gifted grade-matched group on R-CMAS scores. Nevertheless, they tended to exhibit higher scores on the worry/overexcitability scale than the non-gifted age-matched (5th grade) group. Also, they showed the same level of perfectionism as their age peers (in all CAPS scores) and a higher level of perfectionism than their grade peers (in the total CAPS score and in the self-oriented dimension). In conclusion, the gifted children were closer to their grade level peers than to their same-age peers in some internalizing behaviors.

Kermarrec et al. [38] found that children with IQ ≥ 130 compared to children with IQ < 130 showed more anxiety disorders, which mainly included generalized anxiety disorder, phobic anxiety disorder and separation anxiety disorder (ICD-10), diagnosed by the child psychiatrist. Moreover, the authors found that children with verbal comprehension (VCI) scores ≥ 130 described themselves as more anxious on the R-CMAS (total anxiety, physiological anxiety and worry/oversensitivity scores) than children with lower IQ scores. In contrast, children with perceptual reasoning (PRI) scores ≥ 130 stated themselves as less anxious. This study raised the issue that different domains of excellence (either verbal or non-verbal) might have other behavioral correlates in daily life.

Shaywitz et al. [28] found higher scores on the negative affect scale of the Yale Children’s Inventory (YCI; [59]) (e.g., rejection by peers, depression, pessimism) in the gifted group compared to the non-gifted group of children, both groups including boys only. Interestingly, in this study, differences between “low-gifted” and “high-gifted” children were investigated. Moreover, a group with learning disabilities was added. The low-gifted group (IQ range: 124–139) presented a behavioral pattern similar to that one of the non-gifted group on all behavioral measures, whereas, the high-gifted group (IQ range: 140–154) presented a behavioral pattern similar to that one of the children with learning disabilities, characterized by hyperactivity and distractibility. When comparing low-gifted (IQ range: 130–145) vs. high-gifted (IQ ≥ 145) children, Guénolé et al. [29] found that the first group did not show more internalizing symptoms (16.7%) than the second group (10.3%). However, “low giftedness” was associated with more somatic disorders than “high giftedness”. Beyond the contrasting findings, the two latter studies revealed that the “level” of intellectual giftedness might impact behavior.

Richards et al. [35] examined a group of gifted and a group of non-gifted pre-adolescents and adolescents using the BASC. Teachers’ ratings revealed no significant differences between the groups. On the other hand, parents’ ratings revealed that the gifted group showed lower levels of anxiety and attention problems than the non-gifted group, demonstrating a better overall psychological adjustment. Furthermore, self-report scores of the gifted group on the depression scale and on the sense of inadequacy scale were significantly lower than their average ability peers, indicating better emotional adjustment and less inadequacy, respectively. This study highlights the importance of comparing ratings from multiple observers. In line with this, Eren et al. [33] investigated a wider age-range sample (from 9 to 18 years). They did not find differences in anxiety and depressive symptoms between the gifted and the non-gifted group of children when considering parents’ ratings (K-SADS, SDQ, PedsQL) as well as child’s ratings (K-SADS, SDQ to adolescents, PedsQL, State-Trait Anxiety Inventory for Children [60], Child Depression Inventory [61]) and clinician evaluation (Children Depression Rating Scale-Revised – CDRS-R [62]). On the parent-rated Family Assessment Device [63], family members of gifted children showed more care for each other than the non-gifted group.

In adulthood, Karpinski et al. [26] found that the gifted participants showed an increased rate of mood disorders (mood disorders, bipolar disorder, other mood disorders) and anxiety disorders, such as generalized anxiety disorder and obsessive-compulsive disorder than the national average.

### Externalizing Problems

Few studies (5 out of 19) examined externalizing problems related to giftedness, such as hyperactivity and oppositional behaviors. As specified below, these studies provided inconsistent evidence.

Peyre et al. [30] found no difference in hyperactivity/inattention and conduct problems between gifted and non-gifted preschool children, as rated by parents in the SDQ. Shaywitz et al. [28] found more problems of tractability (e.g., needing constant supervision, difficulty with babysitters and with visiting friends) in high-gifted age-school children compared to low-gifted children and non-gifted children, as reported by parents in the YCI. Eren et al. [33] reported more inattention/hyperactivity problems in the gifted group relative to the non-gifted group, as revealed by the child-rated SDQ. Also, Guénolé et al. [29] showed more externalizing problems (delinquent and aggressive behavior). More specifically, low-gifted children displayed more mixed syndromes than high-gifted children. Interestingly, mean CBCL raw scores between gifted children with and without a significant verbal-performance discrepancy (SVPD) on the Wechsler Intelligence Scale for Children (WISC) were compared. The subgroup of gifted children with SVPD showed higher scores in externalizing problems, aggressive behavior, and mixed syndrome than gifted children without this discrepancy.

In contrast, in a general population-based study (n = 2221) of 10–12 years old children, Rommelse et al. [42] did not find an increased rate of externalizing problems in a gifted group of children and pre-adolescents compared to non-gifted controls, as assessed by the parent-, teacher- and self-rated instruments (i.e., CBCL, Teacher’s Report Form, and Youth Self-Report).

### Comorbidity with ADHD

The association between intellectual giftedness and ADHD symptoms was the most investigated comorbidity (9/19 studies). Six studies compared groups of gifted children with a diagnosis of ADHD with groups of gifted children without ADHD. Chae et al. [31] found that gifted children with ADHD obtained poorer scores on the CBCL social competency scale compared to both gifted children without ADHD and non-gifted children. Unfortunately, this study has a limited sample size of children with ADHD in the gifted group, therefore this comparison should be taken cautiously.

Antshel et al. [36] showed that gifted children with a diagnosis of ADHD were more likely to have psychopathological comorbidities (according to the CBCL scales), to repeat a grade, and to show functional impairments in all social domains (according to the Social Adjustment Inventory for Children and Adolescents, SAICA scales [64]), such as in the relationships with peers, with the opposite sex, and with parents. On the other hand, they carry on significantly more activities in their spare time and with their father. The K-SADS interviews with mothers and with children (older than 12 years) revealed a lifetime history of multiple disorders, namely, depression, generalized anxiety, separation anxiety, social phobia, simple phobia, oppositional defiant, and conduct disorders [36]. The follow-up study [19], conducted 4.5 years later, demonstrated that the differences in psychopathological comorbidities between high-IQ children with ADHD and high-IQ children without ADHD persisted over time, with significantly higher rates of depression, separation anxiety, social phobia, simple phobia, oppositional defiant and obsessive-compulsive disorders. The ADHD status was still a significant predictor of higher functional impairment across social, academic, and family domains. Since the studies of Antshel et al. lacked a non-gifted control group, we compared the average scores of the gifted participants without ADHD with the normative cut-offs [64, 65]. We found that in Antshel et al. [36] they obtained higher scores in all subscales of the CBCL and in the subscales that assess problems in spare time in SAICA. In contrast, in the follow-up study [19], they reported lower scores than the normative mean in all the SAICA subscales (except for boy-girl relationships). This finding could reflect a remission of functional impairments.

Foley-Nicpon et al. [40] compared two age-matched gifted groups of children and adolescents, with and without ADHD. The ADHD group reported lower positive self-esteem (BASC-2), lower self-concept about their behavior, and worse overall happiness (Piers–Harris Self-Concept Scale 2 [66]). On the other hand, they showed neither differences in the perception of relationships with others, intelligence, and physical attributes, nor differences with regards to self-reported symptoms of anxiety, popularity, self-reliance, or social stress.

As high-IQ children with ADHD, high-IQ adults with ADHD reported higher rates of major depressive disorder, generalized anxiety disorder, and obsessive-compulsive disorder, compared to high-IQ adults without ADHD [25]. Moreover, they reported lower quality-of-life ratings (in Quality of Life Enjoyment and Satisfaction Questionnaire, Q-LES-Q [67]) and more functional impairments across a variety of occupational, social and family domains (in Social Adjustment Scale-Self-Report [68]). The authors attributed these symptoms to the stress deriving from high intelligence and a clear understanding of the potential consequences, in the context of impulsivity and lack of executive control. In contrast, other psychiatric comorbidities, such as substance abuse disorders, alcohol abuse, or antisocial personality disorder, did not differ between gifted groups with and without ADHD. When we compared the gifted adults with ADHD of Antshel et al. [25] to similar non-gifted participants with ADHD in another study by Mick et al. [69], we found better ability to get around without dizziness in the first group. On the other hand, the gifted adults without ADHD of Antshel et al. [25] had lower scores on Q-LES-Q in physical health, in the ability to get around without dizziness compared to the study by Mick et al. [69]. Still, they had higher scores in household activities, social relationships, leisure time, living/housing situations, and overall life satisfaction. This finding would suggest that the quality of life in gifted adults with ADHD might be better in some domains, such as social relationships, than in non-gifted adults with ADHD.

A more recent study compared two groups of gifted children, with and without ADHD, to two groups of non-gifted age-matched children, with and without ADHD [39]. In the Strengths and Weaknesses of Attention-Deficit/Hyperactivity Disorder Symptoms and Normal Behavior Scale (SWAN [70]), the two groups without ADHD did not differ in inattention, hyperactivity/impulsivity, and total ADHD scores. Conversely, both gifted and non-gifted groups with ADHD had higher average scores than the gifted and non-gifted groups without ADHD. Notably, the non-gifted group with ADHD reported higher scores than the gifted with ADHD group for all inattention symptoms; the opposite pattern was found for some hyperactivity/impulsivity behaviors (modulation of motor activity, modulation of verbal activity, and reflecting on questions). Compared to non-gifted children with ADHD, children with ADHD may be more often hyperactive than inattentive. Additional investigations comparing gifted children with ADHD with gifted children without ADHD are needed to clarify whether giftedness may attenuate or exacerbate ADHD symptoms.

A separate consideration has to be made for the studies that examine the presence of ADHD symptoms in high-IQ individuals. Rommelse et al. [42] found that the IQ scores in a children population were inversely related to attention and hyperactivity/impulsivity problems. However, high discrepancies among parents’ and teachers’ ratings emerged in cases with extreme IQ values. Namely, parents reported more attention problems in children with high IQ, whereas teachers reported more attention problems in children with low IQ. According to the authors, the type of environment, more or less stimulating, plays a major role in determining attention problems: since the school is a cognitively more stimulating environment than the home, it could elicit fewer attention problems in highly intelligent children.

McCoach et al. [27] investigated the presence of inattention and hyperactivity/impulsivity problems in gifted children and adolescents, according to the ADHD-IV rating scales [71] scored by parents and teachers. Both raters reported significantly greater inattention problems in the gifted compared to the non-gifted group, with greater frequency in the classroom than at home. The gifted children with higher inattention scores were also those with lower scores in goal-valuation, self-efficacy and, most importantly, self-regulation measured by the School Achievement Attitudes Survey-Revised [72]. Furthermore, they showed lower academic grades. In line with Rommelse and coworkers, the authors suggested that context-specific inattention in intellectual giftedness would be attributed to boredom or lack of a stimulating environment, more than to ADHD.

### Comorbidity with ASD

Surprisingly, among the studies included in the present review only one study examined the association with ASD, although this is commonly observed in clinical settings. Specifically, Doobay et al. [37] investigated adaptive and psychosocial functioning in gifted participants with ASD (from 5 to 17 years). A multi-informant instrument was adopted (the BASC-2), and an inter-rater discrepancy emerged across self-assessment, parents’ and teachers’ assessment. According to parents’ reports, the gifted ASD group had significantly higher scores than the gifted non-ASD group on almost all scales (aggression, attention, atypicality, depression, hyperactivity, somatization, and withdrawal). In particular, mean scores on the atypicality and withdrawal were above the clinical cut-off. Also, parents’ ratings on communication, daily living, and socialization skills in the Vineland Adaptive Behavior Scales [73] were significantly worse in the ASD than in the non-ASD group. These results suggested to the authors that the presence of an ASD comorbidity altered the socio-emotional and behavioral patterns in giftedness. Similar results were observed in the teachers’ reports, although no scores above the clinical cut-off were obtained. According to the authors, gifted children/adolescents interacting with adults more than peers is sometimes not deemed as a social deficit, but a sign of maturity, sophistication, or intellect. On the other hand, young intellectually gifted people with ASD may better understand and have more awareness of their psychosocial challenges. Therefore, they may be more able to express and articulate socio-emotional difficulties, such as depression or inattention, than their ASD peers with lower intellectual functioning do. Unfortunately, since a control group of non-gifted children with an ASD diagnosis was missing in the study, the hypothesis that intellectual giftedness might attenuate behavioral problems remains unaddressed. In contrast to parents’ and teachers’ reports, self-reports did not differentiate gifted participants with and without ASD. Moreover, a more positive picture of emotion regulation skills emerged in the first group of participants. Of note, all scores rated by all informants in the gifted non-ASD group were in the normal range. This means that gifted children, adolescents, and teens were no more likely than their peers to suffer social or emotional difficulties.

## Discussion

This study provides a systematic review of empirical research that, in the last twenty years, has examined the presence of socio-emotional and behavioral disorders in intellectual giftedness, to investigate how often giftedness coexists with such disorders. Overall, the results leave no room for an unambiguous answer to this question, since some studies have revealed an association whereas others have not.

### Socio-Emotional Problems

Only one study was detected that investigated differences between gifted and age-matched non-gifted children in the preschool age [30]. According to parents’ reports (in the SDQ), no differences between the gifted and control group in peer relationships and prosocial behaviors emerged in this study. On the other hand, some studies that investigated socio-emotional behaviors in older children revealed the presence of problems. Guénolé et al. [29] found that, according to parents’ reports (in the CBCL), 8–11 years old gifted children have more social problems, withdrawal, and aggressive behaviors than an age- and sex-matched control group. In a similar age range, Guignard et al. [34] found that children self-reported (in the R-CMAS) more social concerns than the age-matched non-gifted group, but not compared to the grade-matched non-gifted group. Of note, this study has the methodological strength that two control groups were enrolled, a first control group, which included non-gifted children who matched the education level, and a second control group, which included non-gifted children who matched the age. Since gifted children tend to be younger than their classmates in the event of an acceleration in the curriculum, this method allows for comparison with both grade level and age peers.

In contrast to the above-mentioned studies, Eren et al. [33] found no differences in parents’ scores on prosocial behavior and peer relationship, when comparing gifted children and adolescents in a wide age range (9 to 18 years) with a non-gifted age- and sex-matched group. In line with this, Richards et al. [35] found no difference between the gifted and the non-gifted group of children (7 to 10 years) in the social skill scale scored by teachers. Instead, they found better socio-emotional adjustment according to the child’s evaluation.

Regarding adolescence, the semi-structured interview K-SADS revealed that gifted adolescents described themselves as more shy and insecure, and as having fear of social situations [41]. Moreover, Chung et al. [32] found that gifted adolescents involved in a game showed more cooperative behaviors toward their peers. Interestingly, this study pointed out that prosocial behaviors were more frequently present in gifted people when these behaviors are rewarded. This suggests that social concerns are not always paralleled by corresponding withdrawal behaviors or might be overcome if social behavior is rewarded.

In adulthood, Karpinski et al. [26] did not find differences in the prevalence of social anxiety diagnosis when comparing a sample of gifted adults with the national average.

Overall, these results raise two important issues. First of all, socio-emotional (and behavioral, as shown below) problems vary according to age, and adolescence might be critical, especially when reports come from the eyes of the adolescent. Only one study investigated socio-emotional and behavioral disorders in gifted children of preschool age [30], and did not find differences compared to the age-matched controls. This could suggest that such problems might emerge or be evident later in age; however, further cross-sectional or longitudinal studies are needed to elucidate the age effect. Second, observations derived from different sources/contexts often do not converge. Therefore, using multi-informant instruments is critical for evaluating each case appropriately.

### Internalizing and Externalizing Problems

The results of the studies investigating internalizing and externalizing problems are inconsistent. When we grouped them according to age, we found that preschool children did not show greater problems than the non-gifted group [30]. Namely, somatic complaints, fear of new situations, depressive or irritable behaviors, conduct problems, and symptoms of hyperactivity/inattention in high-IQ children, rated by parents (in the SDQ), were comparable to children with IQ in the normal range. Similarly, Shaywitz et al. [28] did not find significant differences in internalizing and externalizing symptoms between gifted and non-gifted groups of 9- to 12-year-old boys, according to their parents’ ratings (in the YCI). In contrast, some studies examining school-age children revealed the presence of problems. Parents of gifted children, aged 8 to 11 years, reported more internalizing (somatic complaints, anxiety/depression, thought problems, attention problems) and externalizing (breaking the rules and aggressive behaviors) problems (in the CBCL) than non-gifted age-matched children [29]. Remarkably, the discrepancy between verbal IQ and performance IQ was associated with greater aggressive behaviors and mixed syndrome. Gifted children self-reported greater symptoms of somatic manifestations of anxiety (such as sleep disorders in the R-CMAS) and a tendency to perfectionism (in the CAPS) relative to age-matched and grade-matched non-gifted children, respectively [34]. These results suggest that some internalizing aspects are more sensitive to age development, whereas other aspects are more sensitive to the school setting.

Of note, Guénolé et al. [29] and Shaywitz et al. [28] compared low- and high-gifted children. While Guénolé et al. [29] found a greater prevalence of internalizing and externalizing symptoms in the low-gifted group, whereas very high IQs were not associated with increased behavioral problems, Shaywitz et al. [28] did not find differences between low- and high-gifted groups, and the latter group was more similar to the group of children with learning disabilities in terms of behavioral problems. These contrasting results could be likely attributed to the different recruitment contexts. The children of Guénolé et al. [29] came from a hospital and from the private practice of pediatricians, where they were referred because of socio-emotional problems and/or school underachievement or maladjustment. In contrast, Shaywitz et al. [28] came from special programs/classes for gifted students. Another possible explanation could be the use of different instruments. While Guénolé et al. [29] used a more comprehensive assessment (the CBCL), Shaywitz et al. [28] used an instrument more centered on attention, conduct, and cognitive problems (the YCI). Moreover, Guénolé et al. [29] included both boys and girls, albeit the majority were boys (70%), whereas Shaywitz et al. [28] involved only boys and smaller sample size. Regardless of methodological differences, the results of these studies suggest that the “level” of intellectual giftedness might play a role in determining behavioral problems.

The study of Eren et al. [33] did not find differences in internalizing or externalizing disorder in gifted participants aged 9 to 18 years relative to non-gifted controls. However, when clinicians’ reports (the CDRS-R) were considered, more depressive symptoms emerged in gifted boys compared to gifted girls. Furthermore, children rated themselves (in the SDQ) as more inattentive/hyperactive. Based on the child or adolescent observations provided by the psychiatrist, Kermarrec et al. [38] found a higher prevalence of anxiety disorders in 6- to 11-year-old children with IQ > 130, mainly generalized anxiety disorders. When looking at the parents’ reports, there were no significant associations between IQ scores and anxiety disorders. Interestingly, when looking at the child’s self-evaluation and considering verbal comprehension (VCI) and perceptual reasoning (PRI) indexes separately, it emerged that children with higher scores on VCI reported significantly more anxiety symptoms than non-gifted, whereas children with higher scores on PRI reported significantly fewer anxiety symptoms. The authors proposed that a higher verbal IQ might be considered a risk factor for anxiety disorders, whereas a higher perceptual-reasoning IQ might be considered a protective factor. Taken together, the results of Eren et al. [33] and Kermarrec et al. [38] revealed the importance of considering different raters and verbal and non-verbal abilities separately.

In an adolescent group, Richards et al. [35] found a lower rate of internalizing and externalizing disorders in gifted compared to non-gifted peers, according to parents, teachers, and self-report ratings. Indeed, parents observed better emotional and behavioral adjustments. The finding that gifted students had relatively low levels of depression, high self-reliance, a greater sense of adequacy, and better attention, suggested to the authors that intellectual giftedness rather than being a source of vulnerability is a protective factor. In line with this finding, Rommelse et al. [42] found that attention and hyperactivity-impulsivity problems decreased with increasing IQ scores (from 55 to 145), in 10 to 12 years old children. In contrast, Karpinski et al. [26] found that gifted adults reported more frequent mood, anxiety, and obsessive-compulsive disorders than the national average.

Overall, the studies that examined internalizing and externalizing problems in intellectual giftedness revealed once again the importance of considering the effect of age on symptoms’ manifestation and of using comprehensive instruments (e.g., BASC, CBCL) as well as a multi-informant approach. Psychiatric clinical observations might not converge with parents’ observations, or parent reports do not correspond to teachers’ or self-reports. This is likely due to different perceptions of informants about a given behavior. Indeed, self-report instruments are more sensitive to the inner experiences of the adolescent, whereas parent and teacher ratings take into account overt behaviors. Moreover, it could be in part due to the differential expression of a disorder according to the setting.

Also, the studies’ results raised the issue of the usefulness of evaluating verbal and non-verbal dimensions of intellectual giftedness separately, given that they could be differentially associated with behavioral disorders [38]. More specifically, the discrepancy between verbal and non-verbal intelligence (SVPD) could be related to more severe symptoms [29]. Therefore, it has been hypothesized that an “asynchronous development” [74] of intelligence in different domains exposes children to a higher risk of emotional and behavioral dysregulation.

### Twice-Exceptionality

The presence of twice-exceptionality, such as the comorbidity with ADHD or ASD, was more unequivocally associated with socio-emotional and behavioral problems. The studies that compared gifted participants with a diagnosis of ADHD or ASD with gifted participants without the diagnosis revealed poorer social skills [31, 37]. Furthermore, an ASD diagnosis in giftedness was associated with higher atypicality and withdrawal [37]. Similarly, externalizing problems were more common among gifted participants with ADHD (e.g., destructive behavior disorders [19, 36]) or ASD (e.g., aggression [37]), compared to the gifted groups without ADHD or ASD. In their longitudinal study, Antshel et al. [19, 25, 36] found that, compared to gifted people without ADHD, gifted people with ADHD (children, adolescents, and adults) showed more psychopathological comorbidities (major depressive disorder, generalized anxiety, obsessive-compulsive disorder), which persist over time.

Rommelse et al. [42] argued against the hypothesis that higher IQ scores may increase the risk of ADHD symptoms. They found an inverse relationship between IQ and attention and hyperactivity/impulsivity disorders in children, according to both parents’ and teachers’ reports. In line with a more recent study [27], the authors concluded that the environmental context played a significant role in inattention symptoms, given that they are attenuated in stimulating programs/situations. Fewer symptoms are likely to emerge in richly stimulating and more challenging contexts (school and/or home) [27, 42]. In a poorly stimulating and lacking in challenges context, gifted children may appear to be affected by ADHD, and judged inattentive and impulsive by parents and teachers, when in fact their lack of concentration would be better attributed to boredom and demotivation. Instead, giftedness could act as a protective factor that would compensate for attention problems, providing effective internal control strategies, depending on the task [31].

Given the large overlap between intellectual giftedness, ADHD, and ASD profiles, we might conclude that the assessment of related symptoms in giftedness should be always taken into account. The main weakness of the studies that examined twice-exceptionality is the lack of a control group of non-gifted participants with ADHD or ASD diagnosis, which does not allow to directly test whether intellectual giftedness attenuates or exacerbates the psychopathology severity.

### Final Remarks

From a careful examination of the existing literature, we might conclude that a unique answer cannot address the issue of whether intellectual giftedness implies a higher prevalence of socio-emotional and behavioral disorders as compared to normal-range intelligence. Some studies suggest that the existence of intellectual giftedness may potentially be a risk factor for developing behavioral and socio-emotional disorders. On the other hand, it may sometimes protect against developing them. For example, a high IQ may contribute to finding helpful strategies for coping with difficulties and symptoms (e.g., [35, 42]).

Factors that modulate the expression of symptoms could be the discrepancy between giftedness in verbal and non-verbal domains; age, sex, the context/setting in which the child is observed, and the “level” of intellectual giftedness. The discrepancy between scores on verbal and non-verbal tests in the Wechsler scales was associated with a higher risk of developing comorbid disorders. In particular, children with SVPD (i.e., more than 15 points of difference between the scales) showed more externalizing or mixed behavioral syndromes, with symptoms of a relatively severe nature, which reflect emotional and behavioral dysregulation [29]. On the contrary, internalizing problems were present more often in children with high verbal IQ than in children with high non-verbal IQ [38]. The finding of different profiles in the case of significant discrepancies between the Wechsler scales raises the need to clarify the relationship between the individual cognitive profile, covering several domains, and the variability in behavioral, emotional, and social adaptation. 

The studies discussed here suggest that high-IQ children may not show socio-emotional or behavioral problems until later in childhood. Sex could also play an important role in determining the presence of comorbid problems. Indeed, the study by Eren et al. [33], which was the only one that directly tested sex effects, reported greater social problems (peer relationship problems and social behavior) in gifted vs. non-gifted boys but not in girls. To be corroborated, these findings need further evidence from cross-sectional studies, which would compare age ranges and test sex differences.

One limitation of the studies examined here was that information on the child was often derived from one informant/setting only. Given that environmental conditions might moderate (exacerbate or mask) the expression of behavioral symptoms, the use of multi-dimensional tools that allow crossing reports from multiple informants (parents, teachers, clinicians, child) has emerged as a critical factor for an appropriate assessment of the child.

The analysis of the effect of IQ considered as a continuous variable, from 50 to ≥ 130, was a valuable tool for clarifying the association between IQ and behavior [42], especially if considering that there is no agreement on a unique full IQ cut-off value for intellectual giftedness (as shown in Table 1, the cut-off value could be either 120 or 130). Also, the distinction between “high” and “low” gifted profiles revealed different behavioral patterns [28, 29].

As already raised in two previous reviews [5, 23], a non-gifted control group should be recruited and the recruitment setting should be considered. Indeed, the gifted group is sometimes recruited in clinical settings or from special schools, whereas the control group is recruited among the general population. Therefore, a bias toward observing psychopathology or more significant differences might emerge.

Future studies should address the lack of investigation of comorbidity of ASD and intellectual giftedness, always keeping in mind that a gifted non-ASD group should be added to the non-gifted ASD group. Moreover, being the SVPD a classical feature of Asperger syndrome, it could be hypothesized that a proportion of clinically referred gifted children must be screened for ASD. 

We did not find empirical studies investigating the comorbidity of intellectual giftedness with learning disabilities, although this is observed in clinical practice [75]. This limitation was perhaps due to the difficulty in comparing the performance of gifted individuals in learning disability tests with normative cut-off values based on the average-IQ population.

### Limitations and Future Directions

The main limitation of the examined studies is that the concept of intellectual giftedness was centered on an IQ-based categorization. Sometimes the IQ score was even based on two Wechsler tests only. Although the use of a universal cut-off (e.g., IQ > 130) is necessary as an inclusion criterion, this measure alone is neither exhaustive nor comprehensive to fully understand the relationship between giftedness and the individual emotional, social, and behavioral development. While presenting the same IQ, different children can have extremely different intellectual profiles. The finding that children with higher verbal than non-verbal intelligence may show a socio-emotional and behavioral pattern completely different from children with higher non-verbal intelligence raised the need to consider specific patterns of comorbid disorders for different performance areas. This heterogeneity encourages the use of a wider concept of intelligence, such as the multiple intelligence perspective [3, 4]. More importantly, it suggests that a more appropriate approach should be capitalizing on inter-individual variability in cognitive functioning instead of delineating a unique profile of functioning that encompasses all gifted individuals, as outlined by a recent review on neuropsychological functioning in high IQ individuals [76].

While future studies should certainly disambiguate methodological confounds, some theoretical rethinking is needed to elucidate the heterogeneous nature of intellectual giftedness and to grasp the nature of the coexistence of high scores on intelligence tests and psychopathological behaviors.

## Summary

The evidence provided by the studies examined in this review does not solve the question of whether intellectual giftedness is associated with socio-emotional and/or behavioral problems. It can be both a risk factor and a protective factor that would compensate for the evolution of disorders. Overall, it emerges the need of research to adequately assess comorbidity in giftedness, considering that sometimes behavioral manifestations may depend on factors/moderators, such as age, the more or less stimulating context/setting in which the gifted individual is educated, and the rater (parent, teacher, clinician, or self). Importantly, the discrepancy between verbal and non-verbal IQ seems to be the factor most associated with poor emotional development. On the other hand, the presence of giftedness seems to protect against more severe inattention disorders. Whether intellectual giftedness is associated with socio-emotional and/or behavioral problems more often than the non-gifted population is still an open question, on which we encourage future studies.

### Electronic supplementary material

Below is the link to the electronic supplementary material.

Supplementary Material 1
