# Supplementary material for: Behavioral and Socio-Emotional Disorders in Intellectual Giftedness: A Systematic Review
Source: Child Psychiatry Hum Dev. 2022 Oct 1;55(3):768–89. doi: 10.1007/s10578-022-01420-w (PMC11061066; doi:10.1007/s10578-022-01420-w)
Supplement: Supplementary file 1 — Supplementary Material 1 [file 10578_2022_1420_MOESM1_ESM.docx]

**Supplementary material**

**Cognitive measures**

The Wechsler’s intelligence scales have been used for the general cognitive assessment and the measurement of IQ (see Table S1). Specifically, the following test batteries were administered: the WPPSI-III (Wechsler Preschool and Primary Scale of Intelligence 3rd Edition [1]), the WISC-R (Wechsler Intelligence Scale for Children [2]), the WAIS-III (Wechsler Adult Intelligence Scale 3rd Edition [3]**)**, the WISC-III [4], and the WISC-IV [5] in more recent studies [6–10]. In the case of WPPSI-III, WISC-R, WISC-III, and WAIS-III the full IQ score is based on the scores on verbal (Verbal IQ, VIQ) and performance (Performance IQ, PIQ) scales, while in the case of WISC-IV the full IQ score is based on four indices: Verbal Comprehension (VCI), Perceptual Reasoning (PRI), Working Memory (WMI), and Processing Speed (PSI). In one study [11], alternative general reasoning tests (e.g., the Jenkins NonVerbal Group test [11] or the Stanford Binet [12]) were applied to provide an IQ score.

In addition to the Wechsler’s scales, other tests were performed for estimating individual academic strengths and weaknesses (i.e., the Woodcock-Johnson test, WJII [13, 14]), reading and arithmetic skills (i.e., the Wide Range Achievement Test, WRAT-R [15–18]), silent reading test for text comprehension [19], and non-verbal reasoning (i.e., the Cattell's Culture Fair Test [19, 20]).

The chosen cut-off for the “giftedness” was in almost all studies an IQ score ≥ 130 on at least one scale of the Wechsler battery or on the other alternative tests. Instead, in one study the chosen cut-off was an IQ score ≥ 127 [11], and in four studies an IQ score ≥ 120 [12, 15–17]. The specific cut-off values for each study are reported in Table 1.

**Behavioral measures**

For the behavioral assessment, we report here a brief description of the tools used in the studies reviewed in this work.

The Child Behavior Checklist (CBCL [21]) is one of the most used instrument and assesses the presence of behavioral symptoms that commonly occur from preschool age to 18 years. Typically, it is administered to parents, but it has also a teacher (the Teacher's Report Form, TRF) and a self-report form for children older than 6 (the Youth Self-Report, YSR). The CBCL subscales are grouped in those that investigate “internalizing” symptoms (anxiety/depressed behaviors, withdrawn, somatic complaints), those that investigated “externalizing” symptoms (rule-breaking/delinquent behaviors and aggressive behaviors), and those that investigate social, thought and attention problems.

The Behavior Assessment System for Children (BASC [22], BASC-2 [23]) is a multi-dimensional scale applicable to children/adolescents aged 2 to 21 years, addressed to parents, teachers, and self. It assesses externalizing (aggression, hyperactivity, conduct problems), internalizing (anxiety, depression, somatization), attention, learning (for teachers), atypicality, and withdrawal problems. Furthermore, it includes adaptive scales (for teachers) that assess leadership, social skills and study skills. The self-report measures, obtained from children older than 12 years, are divided in clinical maladjustment (anxiety, atypicality, locus of control, social stress, somatization), school maladjustment (attitude to school, attitude to teachers, sensation-seeking), and personal adjustment (relations with parents, interpersonal relations, self-esteem and self-reliance).

The Yale Children's Inventory (YCI [24]) is a multi-dimensional parent-based rating scale, which measures child's behavior, attention and cognitive characteristics, with particular emphasis on attention deficits. The examined behaviors are activity, impulsivity, tractability, conduct disorder-socialized, conduct disorder-aggressive and negative affect, whereas the cognitive/academic domains are language, habituation, attention and fine motor skills.

The Strengths and Difficulties Questionnaire (SDQ [25]), completed by parents, teacher and/or children (3-16 years), measures pro-social behaviors, emotional symptoms, conduct problems, inattention/hyperactivity symptoms and peer relationship/problems.

The Vineland Adaptive Behavior Scales (Vineland-II [26]) can be administered to parents either as a semi-structured interview or as a rating form. This tool evaluates the adaptive behavior, that is the activities (communication, daily living skills, socialization and motor skills) habitually carried out in order to respond to the expectations of personal autonomy and social responsibility, typical in people of the same age and cultural background.

The Schedule for Affective Disorders and Schizophrenia for School Age Children, Present and Lifetime Version (K-SADS-PL [27]), is a semi-structured diagnostic interview that investigates the presence of multiple psychopathologies (e.g., depression and ADHD) according to DSM-IV criteria. It integrates parent and child (from 6 to 18 years) responses, allows the comparison of their responses and prompts querying of incongruences.

The Children Depression Rating Scale Revised (CDRS-R [28]) is used to specifically diagnose depression, to determine its severity and the treatment response. It includes 17 symptom areas that cover the criteria necessary for a diagnosis of depression according with the DSM-IV, and is rated by a clinician via interviews with the child and parents.

For the assessment of social/adaptive functioning in multiple domains, the Social Adjustment Inventory for Children and Adolescents (SAICA [29]) has been used, namely, a semi-structured interview of clinician to child (from 6 to 18 years) or parents that provides an evaluation of social behaviors at school, in spare-time activities, in peer relationship, and at home (with siblings and parents). For older ages (≥ 17 years), the Social Adjustment Scale-Self-Report (SAS-SR [30]) was adopted, which evaluates the ability to adapt to own social role (instrumental or expressive) in different domains: at work (as a worker, as a homemaker, as a student), in social and leisure activities, and in family (extended family, marital, parental, family unit).

For the evaluation of anxiety, the Revised Children and Adolescent Manifest Anxiety Scale (R-CMAS [31]) has been used, a self-report inventory that assesses the level and nature of anxiety through three subscales (physiological anxiety, worry/oversensitivity, and social concerns/concentration), in children and adolescent aged 6 to 19. Alternatively, two self-reports instruments were used, the State-Trait Anxiety Inventory for Children (STAI-C [32]), which specifically investigates short-term (state scale) or long-term (trait scale) anxiety status, and the Child Depression Inventory (CDI [33]), which measures the severity of depressive symptoms in children and adolescents between 6 and 17 years of age.

Perfectionism was assessed with the Children and Adolescent Perfectionism Scale (CAPS [34]), a self-assessment tool of two dimensions, self-oriented and socially prescribed perfectionism.

The Strengths and Weaknesses of Attention-Deficit/Hyperactivity Disorder Symptoms and Normal Behavior Scale (SWAN [35]) is a multi-informant rating scale focused on the severity of ADHD symptoms in children under the age of 18 years.

In order to measure the general quality of life, the self- and parent-reported Pediatric Quality of Life Inventory (PedsQL [36]) has been used, which investigates four aspects: physical, emotional, social, and school functioning, from 2 to 18 years. To measures the degree of enjoyment and satisfaction in various areas of daily functioning, in adults, the Quality of Life Enjoyment and Satisfaction Questionnaire (Q-LES-Q [37]) has been used. The Piers–Harris Self-Concept Scale 2 (PH-2 [38]) is a self-report questionnaire to assess self-concept in children and adolescents, from 7 to 18 years. This tool contains six scales covering various aspects of self-concept (i.e., behavioral adjustment, intellectual and school status, physical appearance and attributes, freedom from anxiety, popularity, and happiness and satisfaction) and two validity scales.

In some studies, the child’s attitude toward school was also evaluated. The School Achievement Attitudes Survey-Revised (SAAS-R [39]) investigates the possible cause of underachievement students (from 14 to 19 years). This questionnaire consists of five factors considered to be reasons for student underperformance, including academic self-perception, attitudes towards teachers and attitudes towards school, goal-valuation and motivation/self-regulation. The School Refusal Assessment Scale Revised (SRAS-R [40, 41]) evaluates the symptoms of school refusal disorder (from 6 to 17 years) and identifies their reasons for avoiding school through the four domains: avoiding school-related stimuli that cause negative affect (e.g., teacher), escaping from aversive social or evaluative situations, obtaining attention from others, pursuing positive tangible reinforcement outside of school (e.g., play).

The Family Adaptability and Cohesiveness Evaluation Scale III (FACES III [42]) assesses adaptation and cohesion of the family, such as the degree of separation or connection of family members. It is based on 16 family models resulting from the combination of four cohesion parameters and four adaptability parameters, both scored from low to high. The Family Assessment Device (FAD [43]) assesses structural and organizational properties of families and the patterns of transactions among family members. It consists of a General Functioning scale and six subscales (Problem solving, Communication, roles, Affective responsiveness, Affective involvement, and Behavior control), which identify six dimensions of family functioning.

One study [44] differs from all the others for the use of a game (the Public goods game, PG) to assess social interactions. In particular, it investigates cooperative and free-riding behaviors. At the beginning of the game, the participants are awarded a sum of money which can be invested in a public account or in a private account. Then, participants have to make a choice between cooperative or free-ride behaviors in three different conditions, which promote different levels of reward for cooperation.

**Table S1.** Cognitive assessment for participants’ selection.

| **AUTHORS** | **COGNITIVE ASSESSMENT** | **OUTCOMES** |
| --- | --- | --- |
| Antshel et al. (2007) | WISC-R; WISC-III; WRAT-R. | Mean Full IQ (based on Vocabulary and Block Design scores): Gifted group = 125.8 (SD = 4.2), Gifted group with ADHD = 126 (SD = 4.3). Mean WRAT-R Reading: Gifted group = 116.8 (SD = 9.8), Gifted group with ADHD = 116.1 (SD = 11.4). Mean WRAT-R Math: Gifted group = 114.8 (SD = 14.3), Gifted group with ADHD = 108.6 (SD = 14). |
| Antshel et al. (2008) | WISC-R; WISC-III; WAIS-III; WRAT-R. | Mean Full IQ (based on Vocabulary and Block Design scores): Gifted group = 127.1 (SD = 3.2), Gifted group with ADHD = 125.7 (SD = 3.3). Mean WRAT-R Reading: Gifted group = 115.1 (SD = 6.5), Gifted group with ADHD = 114.9 (SD = 10). Mean WRAT-R Math: Gifted group = 116.4 (SD = 12.2), Gifted group with ADHD = 109.5 (SD = 11). |
| Antshel et al. (2009) | WAIS-III; WRAT-R. | Mean Full IQ (based on Vocabulary and Block Design scores): Gifted group = 127.9 (SD = 6.2), Gifted group with ADHD = 127.9 (SD = 7.5). Mean WRAT-R Reading: Gifted group = 112.9 (SD = 5.1), ADHD Gifted group = 112.4 (4.9). Mean WRAT-R Math: Gifted group = 114 (SD = 9.3), Gifted group with ADHD = 107.7 (11.9) |
| Chae, Kim, & Noh (2003) | WISC-R | Mean Full IQ: Gifted group = 138.2 (SD = 8.29); Gifted group with ADHD = 140.3 (SD = 10.92); Non-gifted IQ range = 83-127. |
| Chung et al., (2011) | WISC-III | Mean Full IQ: Gifted group = 142.6 (SD = 5.95), Non-gifted group = 111.1 (SD = 13.01). |
| Doobay, Foley-Nicpon, Ali, & Assouline (2014) | WISC-IV or WAIS-III. | Mean Full IQ (on at least one Index of the WISC-IV/WAIS-III): Gifted group with ASD = 124.90 (SD = 9.96), Gifted group = 127.88 (SD = 9.31). |
| Eren, Cete, Avcil, & Baykara (2018) | WISC-R | Non-gifted IQ range = 90-109. |
| Guénolé et al. (2013) | WISC-III | Mean Full IQ: gifted groups = 138.6 (range = 130–160). |
| Guignard, Jacquet, & Lubart (2012) | Wechsler’s scales (unspecified edition) | Not specified. |
| Karpinski et al. (2018) | Mensa's Tests | Not specified. |
| Kermarrec, Attinger, Guignard, & Tordjman (2020) | WISC-IV | Mean Full IQ: Gifted group = 139.3 (SD = 6.7), Non-gifted group = 116.1 (SD = 10.5). |
| Lacour & Zdanowicz (2019) | WISC-IV | Not specified. |
| McCoach, Siegle, & Rubenstein (2020) | Stanford Binet LM; WISC-III. | Mean Full IQ (either Stanford Binet LM or WISC-III): Gifted group = 131.8 (SD = 9.2). |
| Peyre et al. (2016) | WPPSI-III | Mean Full IQ: Gifted group = 134.6 (SD = 3.1), Non-gifted group = 103.1 (range = 70-130), Group with disability = 59.7 (SD = 9.1) (IQ <70). |
| Richards, Encel, & Shute (2003) | Test of general reasoning ability (Jenkins NonVerbal Group test or Intermediate G group assessment or ACER MLMQ test of language and mathematics). | Non-gifted IQ range = 97-102. |
| Rommelse et al. (2017) | WISC-R | Full IQ (based on Vocabulary and Block Design scores) ranges: 55-70 (n = 65); 70-85 (n = 318); 85-100 (n = 805); 100-115 (n = 721); 115-130 (n = 268); 130-145 (n = 39). |
| Shaywitz et al. (2001) | WISC-R and achievement test  (Woodcock-Johnson Psychoeducational Battery: Reading,  Math,and Written Language). | Mean Full IQ: “low gifted” group = 133.2 (range = 124-139), “high gifted” group = 145.6 (range = 140-154), learning disabilities group = 116.6 (range = 100-135); non-gifted group = 97.7 (range = 75-126). |

**References**

1. Wechsler D (2002) Wechsler Preschool and Primay Scale of Intelligence - 3rd edition (WPPSI-III) Technical and Interpretive Manual. The Psychological Corporation, San Antonio, TX

2. Wechsler D (1974) Manual for the Wechsler intelligence scale for children, revised. The Psychological Corporation, San Antonio, TX

3. Wechsler D (1997) WAIS-III: Wechsler Adult Intelligence Scale - 3rd edition. The Psychological Corporation, San Antonio, TX

4. Wechsler D (1991) Wechsler Intelligence Scale for Children–3rd edition manual. Psychological Corporation, San Antonio, TX

5. Wechsler D (2003) Wechsler intelligence scale for children–Fourth Edition (WISC-IV). The Psychological Corporation, San Antonio, TX

6. Doobay AF, Foley-Nicpon M, Ali SR, Assouline SG (2014) Cognitive, adaptive, and psychosocial differences between high ability youth with and without autism spectrum disorder. J Autism Dev Disord 44:2026-2040. <https://doi.org/10.1007/s10803-014-2082-1>

7. Kermarrec S, Attinger L, Guignard J-H, Tordjman S (2020) Anxiety disorders in children with high intellectual potential. BJPsych Open 6:E70. <https://doi.org/10.1192/bjo.2019.104>

8. Gomez R, Stavropoulos V, Vance A, Griffiths MD (2020) Gifted Children with ADHD: How Are They Different from Non-gifted Children with ADHD? Int J Ment Health Ad 18:1467-1481. <https://doi.org/10.1007/s11469-019-00125-x>

9. Foley-Nicpon M, Rickels H, Assouline SG, Richards A (2012) Self-esteem and self-concept examination among gifted students With ADHD. J Educ Gifted 35:220–240. <https://doi.org/10.1177/0162353212451735>

10. Lacour AG, Zdanowicz N (2019) IQ over 130 and phobia: Correlation, consequences and other psychopathologies. Psychiat Danub 31:386-389.

11. Richards J, Encel J, Shute R (2003) The Emotional and Behavioural Adjustment of Intellectually Gifted Adolescents: A multi-dimensional, multi-informant approach. High Abil Stud 14:153-164. <https://doi.org/10.1080/1359813032000163889>

12. McCoach DB, Siegle D, Rubenstein LDV (2020) Pay Attention to Inattention: Exploring ADHD Symptoms in a Sample of Underachieving Gifted Students. Gifted Child Quart 64:100-116. <https://doi.org/10.1177/0016986219901320>

13. Shaywitz SE, Holahan JM, Freudenheim DA, et al (2001) Heterogeneity within the gifted: Higher IQ boys exhibit behaviors resembling boys with learning disabilities. Gifted Child Quart 45:16-23. <https://doi.org/10.1177/001698620104500103>

14. Woodcock RW (1977) Woodcock-Johnson Psycho-educational Battery, Part II. Tests of Achievement Teaching Resources, Allen, TX

15. Antshel KM, Faraone S V, Stallone K, et al (2007) Is attention deficit hyperactivity disorder a valid diagnosis in the presence of high IQ? Results from the MGH Longitudinal Family Studies of ADHD. J Child Psychol Psyc 48:687-694. <https://doi.org/10.1111/j.1469-7610.2007.01735.x>

16. Antshel KM, Faraone SV., Maglione K, et al (2008) Temporal stability of ADHD in the high-IQ population: Results from the MGH longitudinal family studies of ADHD. J Am Acad Child Adolesc Psychiatry 47:817-825. <https://doi.org/10.1097/CHI.0b013e318172eecf>

17. Antshel KM, Faraone SV., Maglione K, et al (2009) Is adult attention deficit hyperactivity disorder a valid diagnosis in the presence of high IQ? Psychol Med 39:1325-35. <https://doi.org/10.1017/S0033291708004959>

18. Jastak S, Wilkinson GS Wide Range Achievement Test–Revised 1984. Jastak Associates, Wilmington, DE

19. Guignard JH, Jacquet AY, Lubart TI (2012) Perfectionism and anxiety: A paradox in intellectual giftedness? PLoS ONE 7:e41043. <https://doi.org/10.1371/journal.pone.0041043>

20. Cattell RB, Cattell AKS (1959) Cattell culture fair intelligence test. Bobbs-Merrill, Indianapolis, IN

21. Achenbach TM (1991) Manual for the Child Behavior Checklist/4-18 and 1991 profile. ASEBA, University of Vermont, Department of Psychiatry, Burlington, CA

22. Reynolds CR, Kamphaus RW (2002) The clinician’s guide to the Behavior Assessment System for Children (BASC). Guilford Press 9:234-236. https://doi.org/10.1076/chin.9.3.234.16453

23. Raynolds CR, Kamphaus RW (2004) Behavior assessment system for children: Manual. American Guidance Service, Circle Pines, MN

24. Shaywitz SE, Schnell C, Shaywitz BA, Towle VR (1986) Yale Children's Inventory (YCI): an instrument to assess children with attentional deficits and learning disabilities. I. Scale development and psychometric properties. J Abnorm Child Psychol 14:347-364. https://doi.org/10.1007/BF00915431.

25. Goodman R, Meltzer H, Bailey V (2003) The Strengths and Difficulties Questionnaire: a pilot study on the validity of the self-report version. Int Rev Psychiatry 15:173-177. https://doi.org/10.1007/s007870050057

26. Sparrow SS, Cicchetti D V, Balla DA (2005) Vineland adaptive behavior scales:(Vineland II), survey interview form/caregiver rating form. Pearson Assessments, Livonia, MN

27. Orvaschel H, Puig-Antich J (1987) Schedule for affective disorder and schizophrenia for school-age children: Epidemiologic version: Kiddie-SADS-E (K-SADS-E). Nova University, Center for Psychological Studies, Fort Lauderdale, Fla

28. Poznanski EO, Mokros HB (1996) Children’s depression rating scale, revised (CDRS-R). Western Psychological Services, Los Angeles, CA

29. John K, Gammon DG, Prusoff BA, Warner V (1987) The Social Adjustment Inventory for Children and Adolescents (SAICA): Testing of a new semistructured interview. J Am Acad Child Psy 26:898-911. https://doi.org/10.1097/00004583-198726060-00015

30. Weissman MM, Bothwell S (1976) Assessment of social adjustment by patient self-report. Arch gen psychiat 33:1111-1115. [https://doi.org/10.1001/archpsyc.1976.0177009](https://doi:10.1001/archpsyc.1976.0177009)

31. Reynolds CR, Richmond BO (1985) Revised children’s manifest anxiety scale (RCMAS). Manual. Western Psychological Services, Los Angeles, CA

32. Spielberger CD, Edwards CD (1973) State-trait anxiety inventory for children: STAIC: How I feel questionnaire: Professional manual. Consulting Psychologists Press, Palo Alto, CA.33. Kovacs M (1985) Children’s depression inventory (CDI). Psychopharmacol Bull 21:995–998.

34. Flett GL, Hewitt PL (2002) Perfectionism and maladjustment: An overview of theoretical, definitional, and treatment issues. In: Flett GL, Hewitt PL (eds) Perfectionism: Theory, research, and treatment pp 5-13. American Psychological Association, Washington, DC. [https://doi.org/10.1037/10458-001](https://doi:10.1037/10458-001)

35. Swanson J, Schuck S, Mann M, et al (2006) Categorical and dimensional definitions and evaluations of symptoms of ADHD: The SNAP and SWAN rating scales. [Int J Educ Psychol Assess](https://www.ncbi.nlm.nih.gov/pmc/articles/PMC4618695/) 10:51-70.

36. Varni JW, Seid M, Rode CA (1999) The PedsQLTM: measurement model for the pediatric quality of life inventory. Med Care 37:126-139. https://doi.org/10.1097/00005650-199902000-00003

37. Endicott J, Nee J, Harrison W, Blumenthal R (1993) Quality of Life Enjoyment and Satisfaction Questionnaire: a new measure. Psychopharmacol Bull 29:321-326.

38. Piers E V, Herzberg DS (2002) Piers-Harris 2. Piers-Harris Childrens Self- Concept Scale (2nd ed). Western Psychological Services, USA

39. McCoach DB, Siegle D (2003) The school attitude assessment survey-revised: A new instrument to identify academically able students who underachieve. Educ Psychol Meas 63:414-429. <https://doi.org/10.1177/0013164403063003005>

40. Kearney CA (2002) Identifying the function of school refusal behavior: A revision of the School Refusal Assessment Scale. J Psychopathol Behav Assess 24:235-245. <https://doi.org/10.1023/A:1020774932043>

41. Brandibas G, Jeunier B, Gaspard JL, Fouraste R (2001) Evaluation des modes de refus de l’école: validation française de la SRAS (School refusal assessment scale) [Evaluation of schoolrefusal modes: French validation of the SRAS (school refusal assessment scale)].

42. Joh JY, Kim S, Park JL, Kim YP (2013) Relationship between family adaptability, cohesion and adolescent problem behaviors: curvilinearity of circumplex model. Korean J Fam Med 34:169. [https://doi.org/10.4082/kjfm.2013.34.3.169](https://doi:10.4082/kjfm.2013.34.3.169)

43. Epstein NB, Baldwin LM, Bishop DS (1983) The McMaster family assessment device. J marital fam ther 9:171-180. <https://doi.org/10.1111/j.1752-0606.1983.tb01497.x>

44. Chung D, Yun K, Kim JH, et al (2011) Different gain/loss sensitivity and social adaptation ability in gifted adolescents during a public goods game. PLoS ONE 6:e17044. <https://doi.org/10.1371/journal.pone.0017044>
